# Varying Degrees of Animal Reification by Stakeholders in Experimental Research

**DOI:** 10.3390/ani12020190

**Published:** 2022-01-13

**Authors:** Jacques Cabaret, Ludivine Fortin

**Affiliations:** 1 UMR Infectiologie et Santé Publique, INRAE & University of Tours, 37380 Nouzilly, France; traumerine@yahoo.fr; 2 UFR 10 Philosophy, Ethique Appliquée et Responsabilité Sociale et Environnementale, University Paris 1 Sorbonne, 75005 Paris, France

**Keywords:** experimental animals, reification, animal caretaker, ethics, researchers

## Abstract

**Simple Summary:**

Several key stakeholders are involved in animal research, each with distinct responsibilities and objectives. The animal caretakers, for example, have daily contact with the animals and attend to the practical aspects of their nutrition, sanitation, and health and welfare monitoring. Research scientists can have comparatively limited contact with the animals, with their focus rather orientated towards obtaining robust experimental data. The term reification refers to the treatment of animals as objects for our own use. Amongst stakeholder in experimental research, various rationales exist that may contribute towards the reification of animals. For example, one that is potentially shared by many of the stakeholders is that the benefits of animal research (knowledge and real-life applications) outweigh the potential harms (suffering or restraining of animals). For some animal caretakers, establishing bonds with the experimental animals may be considered unprofessional. For researchers, consideration for the animals may be reduced to cases included in the experiment. Despite the potential for animal reification, it is partly mitigated in this context by the commonly held belief that animal suffering should be reduced as much as possible.

**Abstract:**

The attitude towards animals in research depends on both the role of the stakeholder and their personal characteristics. Most studies on the subject have been carried out on stakeholders from biomedical research institutes with comparatively few sociological studies on stakeholders from agricultural research centers. Previous findings suggest that animal caretakers at agricultural research centers felt undervalued by the hierarchy, and that animal reification was present in the sector. This may indicate that a lack of consideration for the animal subjects correlates with an inadequate sensitivity towards humans. Since these findings were published twenty years ago, there has been an increasing emphasis on the importance and actions of ethics committees in research, animal welfare bodies, and public concern for animals, which may have impacted the current perspective. To better understand current degrees of animal reification amongst stakeholders of agricultural research, we conducted semi-directive interviews at a leading agricultural research institute in France (INRAE). The interviews targeted both animal caretakers and researchers who were involved in the study of infectious diseases in livestock, or the behavior of horses and quails. After having transcribed the recorded interviews into text, semi-automatized analyses were carried out to categorize them into distinct groups, from which the most characteristic words and sentences were extracted. Three groups of stakeholders were identified: (i) animal caretakers involved in invasive infectious disease research; (ii) animal caretakers involved in behavioral research; and (iii) researchers. The findings show that animal caretakers felt acknowledged by their hierarchy. It is possible the increased skill criteria for people recruited into this position over the years, combined with greater prospects for continuous learning and development in the profession, may have fostered a more respectful regard across the hierarchy. The animal caretakers clearly expressed that their primary objective was to successfully execute the research protocols and that the animals were viewed as prototypes for research, with which they could, on occasion, develop a bond with. The bond was more important for animal caretakers involved in behavioral studies than for those involved in the study of infectious diseases, where invasive biological sampling and restraining of the animals is required. Researchers prioritized the procurement of robust data to test hypotheses, analyze phenomena, and publish their results. Their concern for the animals rather reflected the views of the general public opposed to thought-out personal opinions on the matter; this is possibly due to their comparatively limited interaction with the animals. They considered the animals in abstract terms that were indicative of reification. This study concludes that animal reification is still present, albeit to varying degrees amongst the stakeholders.

## 1. Introduction

Animal experimentation has historically played an important role in the development of agricultural and biomedical research. It has also given rise to heated public and philosophical discussion, based chiefly on the moral status of animals. The bygone view of animals as ‘machine-like’ beings was used to justify animal experimentation in the past, but a pivotal shift in the moral consideration and biological understanding of animals in the eighteenth century, and then again in the twentieth century, questioned the use of sentient animals in science, spurring on the animal liberation movement and paving the way for more humane scientific practices [1]. As a result, in 1959, Russell and Burch presented three principles for the use of animals in research, known as the three Rs [2]: (i) replacement (the use of humane alternatives such as non-animal technologies to partly replace animals); (ii) reduction (ensuring that the minimum number of animals is used to answer the scientific question with effective experimental design and statistical analyses); and (iii) refinement (reducing suffering and improving welfare throughout the animals’ lives). An additional R, remember, has consequently been proposed to memorialize and acknowledge the past use of animals in research [3]. To meet the principles proposed by the 3 Rs, local ethics committees were created in many countries to assess the conduct of experimental research projects using animals. However, consistency in the outcomes of different ethics committees could vary depending on the distinct views of the individual committee members [4]. There is also a need to harmonize the guidelines under which they operate, where ethics committees based in countries with more advanced animal regulation systems may face greater external pressures from societal concern [5]. Further, many of the considerations currently in place were intended for laboratory animals used in biomedical research and may not adequately encompass the needs of livestock animals in the USA [6]. However, in Europe, the regulation of all experimental animals is presented in the European Union Directive 2010/63 on the protection of animals used for scientific purposes. Animal welfare bodies were created by this directive to strengthen the actions of the ethics committees in controlling the use of animals in research.

A bond between humans and animals in research has frequently been reported [7] and is not just limited to cases involving primates and companion animals but extends to include less expected species, such as chickens and reptiles. However, some stakeholders in this domain had reportedly been trained to minimize or avoid such interactions with the animal subjects for the sake of objectivity [8,9]. Currently, compassion for experimental animals is rather encouraged [10], and advertised animal caretaker jobs often expect the candidate to have both the inclination and the ability to interact with the animals. The public attitude towards the use of animals in research depends on several factors, such as their personal and cultural position, the animal species in question, and the type of research being carried out [11]. For example, regarding personal factors, females and persons with previous animal experience and an urban background are more likely to object to animal research [11]. People are also less supportive of research that uses animals that could be classified as pets or as having supposedly greater mental aptitudes, although research on rodents is seemingly more accepted. Generally, the use of animals to evaluate toxicity, therapeutic drugs, or vaccines to advance human medicine is better accepted than the use of animal testing in cosmetics. Disparate attitudes have also been recorded across the different stakeholder positions in research. For example, researchers are often distanced from the animal subjects [12] and instead prioritize the management of experimental protocols, data analyses and their scientific communication, and securing funding [13]. Such research objectives, with respect to animals, could result in their reification. According to [14], reification means that the properties of objects, subjects and social relations become “thing-like”. Few sociological records exist concerning animal reification by stakeholders involved in experimental research, and those that are available are focused on stakeholders from biomedical research institutes [13].

Animal caretakers have comparatively high levels of contact with the animals, but the literature suggests that, despite their practical skills and knowledge, they feel their contribution is not fully acknowledged by the researchers [13]. A similar lack of acknowledgment has been recorded for laboratory technicians in medical research [14,15]. This corresponds to the theory of recognition (or its absence) by Honneth [16], who defined reification as the “forgetting” of recognition under two aspects: recognition of the other and of the self. “The individual must manifest a kind of emotional self-recognition, and this recognition is nothing other than “self-love”. *Without this emotional recognition, we contemplate our mental states only as facts without value, or we produce states conforming to an external demand, which amounts to producing them in a purely instrumental way*” [16]. Thus, animal caretakers may experience reification at two different levels: from the researchers and from themselves, which may affect a lack of meaning in their work [17]. The reification of animals is also related to the reification of their caretakers: “*Animal research… not only reifies animals but also the human persons involved in this process*” [18]. The present work aimed to test the hypothesis of animal reification amongst caretakers and researchers. We focused specifically on stakeholders involved primarily with livestock animals, based at a leading agricultural research institute in France. Both classical and computerized methods were applied to analyze the interviews to provide complementary information from the interviews.

## 2. Materials and Methods

Semi-directive interviews were carried out in French with stakeholders from the French Agronomical Research Institute and Environment (INRAE) in Nouzilly, France. The interviewers (J.C. and L.F.) asked open questions to stakeholders; these questions had been pre-prepared in an interview guide, which was identical for all interviews. The interviews lasted approximately one hour. The stakeholders interviewed were involved with the research of either the infectious diseases of livestock and/or to a lesser extent, common laboratory animals, or with livestock behavior. The slaughter of any livestock at this research center, either for experimental purposes or market value, were carried out at an on-site abattoir. Semi-directive interviews were selected based on the relative merits of the methodology to gather qualitative empirical research data [19,20]. The stakeholders interviewed had at least four years’ research experience in the field and included researchers, laboratory technicians, animal caretakers, and an abattoir worker. The characteristics of the stakeholders interviewed are presented in Table 1.

The recorded interviews were transcribed into a Word text. Tropes (V8.5) speech analysis software was first used to process the data [21] based on cognitive analysis of the interviews [22] and then analyzed using multivariate methods [22,23] applied to the most frequently used words in the interview. We aimed to identify homogeneous groups amongst the stakeholders, as has previously been performed on horse breeders [24]. Significant differences between homogeneous groups of stakeholders were assessed using Z score statistics for two populations; where the proportions were low (less than 4%), Fisher’s exact test was applied to the number of occurrences for each word. The classical way to interpret interviews was also used: the most exemplary sentences were selected by the interviewers and combined to describe the relationship and work with animals.

## 3. Results

### 3.1. Categorized Groups of Stakeholders Based on Computerized Analysis of Speech

**Figure 1:**
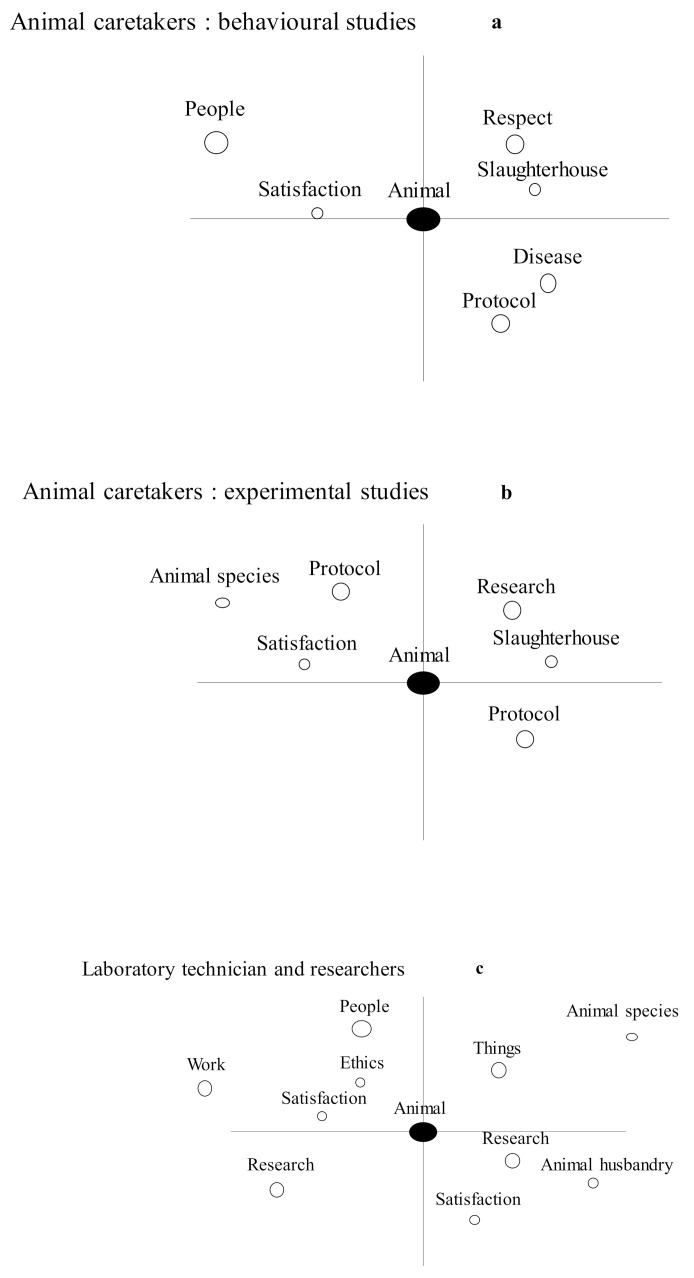
Multivariate analysis of interviews with: (**a**) animal caretakers involved in experimental studies; (**b**) caretakers involved in behavioral studies; and (**c**) laboratory technicians and researchers. The bubble size corresponds to the number of occurrences of the word; the distance between bubbles is smaller when the words are correlated; the left part of the graph corresponds to the beginning of interview and the right part to the end of interview.

Based on the results of the computerized speech analysis, three distinct groups were identified: (i) animal caretakers involved in behavioral research (ACT 5 and 6), (ii) animal caretakers involved the research of infectious disease (ACT 1 to 4), and (iii) a mixed group composed of the abattoir worker, the laboratory technician, and the researchers (R1 to R4). Cognitive-based multivariate analyses showed the words most commonly associated with each of these three groups (Figure 1a–c). Given our interest was in the relationship between the stakeholder and the animals, “animal” was designated as the central word. The left part of the figures presents the words spoken in the beginning of interviews, and the right part presents the words spoken at the end of the interviews. The relative size of the associated circle correlates to the frequency of its use, and its proximity to the central word relates to the intensity of its relationship with the word “animal”.

For animal caretakers involved in behavioral studies, the following traits were identified: they have respect for the animals, they were satisfied with their work, and they were preoccupied with the well-being of their animals (i.e., health, disease, and slaughter in the event of an accident/injury) and in ensuring that the scientific protocol was appropriate to meet the aims. The word ”people” was polysemic (meaning co-workers and other stakeholders or citizens).

For the animal caretakers involved in the research of infectious diseases, the following traits were identified: they were satisfied with their job, their relationship with the livestock animals depended on the species, they accepted that most livestock animals end up at the slaughterhouse; their aim was to follow the research protocol as closely as possible, and they understood the potential application value of their work.

The laboratory technician and the researchers were identified to be satisfied in their work, they were concerned with ethical issues, they had varying attitudes depending on the livestock species under study, and they understood their research was mostly carried out for the advancement of animal health and more efficient husbandry. The word ”people” was again polysemic and could mean colleagues as well as the opinion of citizens.

The multivariate analysis was based on the attitude of stakeholders in relation to the animals. The most frequently used words (>10) by stakeholders from each of the three categories defined above (Figure 1) are presented in Table 2. Significant differences were observed in the occurrence of words spoken by the group that encompassed the laboratory technicians and the researchers and in both groups of animal caretakers (involved in infectious diseases or behavioral studies). These included: a) fewer occurrences of the word “animal”; and b) more occurrences of the words “research and science”, the existence of “problems” to solve, and “suffering/emotions”. Animal caretakers differed between the two research topics. Caretakers involved in behavioral research used the words “animal” and “people” more frequently than the caretakers involved in the research of infectious diseases. Conversely, caretakers involved in the research of infectious diseases used the words “work”, “team”, and “satisfaction” more than the caretakers involved in behavioral research. Some words such as “people” and “work” are polysemic, and their actual meaning can only be deduced after reading their individual interviews.

### 3.2. The Prototypal Animal from the Researcher’s Point of View

After having read transcribed texts of the interviews, the sentences found to be of most common interest among the researchers were extracted. The researchers were aware that the animals should be well treated, and this was reflected in the following statements: “I cannot stand when the animals are mistreated” (R1); “The animals are there for research but they should be treated well… anyway, with the ethics committee, you are guided on what you can and cannot do” (LT1). Researchers also checked how the experimental animals were being kept: “I will come, I will look at the animals… and check if the light is too strong, or if the litter is dirty” (R3). The researchers further specified that their research must meet certain personal and ethical criteria: “A dirty job, for me, and I have never done it, would be something unethical that I could not manage… for example, where horrible things are done to the animals” (R4). Some researchers were found to monitor the animal caretakers’ interactions with the animals: “I think that animal caretakers do not respect the poultry enough… Some animal caretakers do respect the animals, others do not… and for those who do not, you can try hard to send them messages, but you can also see that they are closed to change.” (R3). The researchers’ attitudes towards certain experimental practices may also change with exposure to them, with one researcher stating, “at the beginning, it was difficult for me to kill mice from a batch in order to get organ samples; but after a while, you get used to it, and you do not really see them anymore, you concentrate on the sampling, the results to come” (R2). Their views also depended on the species of animal: “…when people speak of the relationship between man and animals, they generalize it and it disturbs me… animals are plenty, I can’t generalize”; “… a broiler lives for five weeks…you do not have time to see it moving… and it can be difficult to get an idea of the individuals because time is short…with quails you have more time to see them grow” (R3). An R4 researcher indicated that “it looks more professional to show that you are not affected by death or pain of an animal…It is kind of childish to be preoccupied by that”. The researchers’ concern for the animals may extend to the way in which livestock are bred on farms: “I work on farm animals bred in a system which sometimes is not optimal.” (R3).

The researchers were also focused on the ensuing experimental results: “And then to be able to accumulate data, to cross-check with previous data, to have real confidence in what you have seen and observed, this is really pleasant” (R4); “The results are rarely clear cut, so it is very exciting to make sense of them” (R1). Finally, researchers viewed good research “to start with a good question… and then to solve it correctly… to have enough animals involved in the experiment to answer the question… and to have done good work… with the statistical analyses” (R4). The researchers’ point of view could also be self-centered: “when I discover a mechanism… that it works like this… it is a kind of intellectual satisfaction… then we will publish it, well, or not… but that is for external judgment and I am a bit indifferent to it… It is my own personal satisfaction to have understood it that concerns me, nobody shares that” (R3).

### 3.3. Bond and/or Reification of Animals by Animal Caretakers

The relative animal species, the experimental design, and the field of research all had an impact on the caretakers’ relationship with the animals: “Those who work with horses… or work with cattle… they each have a different approach to the animals” (abattoir worker). Animals that spend the majority of their lives in the research center were highly appreciated by some caretakers: “They stay here their whole lives. I would not treat a flock of sheep the same way that only stay for six months” (ACT5); “We don’t assault the animal visually, we don’t assault them physically… It is the basis of the approach. You are not a predator anymore, but the animal is your colleague…you need to be an animal yourself.” (ACT6) Animals that were involved in more intrusive experiments and remained for shorter periods of time were less appreciated: “And a chicken… it doesn’t care about humans. A sheep, when you enter a sheepfold… the animals will look at the human” (ACT1); “Anyway, I speak to all the animals… mostly calves and sheep, but not to chickens.” (ACT2); “Mice are not reactive, they sleep during the day, they are not interesting, but the rats are curious, they are cute” (ACT4).

The animal caretakers involved in the research of infectious diseases had the highest tendency to exclude a real bond with the animals: “I keep my distance with the animals… because I would say… that they are a work tool… same as the buildings where they live, the tractor used for bringing their food”. “If we build a bond, we are going to change our philosophy completely… we won’t be able to have the same contact with the animals… and then, we cannot continue to be their caretaker, it is not possible” (ACT1). Sometimes, this distance may disappear, which can be difficult for the caretaker: “I was performing euthanasia routinely on a batch of mice. Then suddenly, I exchanged looks with a mouse that I was supposed to kill… it was very difficult after this, the mouse had become an individual, I could not kill it and I had to ask a colleague to do it” (ACT4). The view on animals also differed depending on the animal caretaker: “Sheep are all alike” (ACT3); “Take time to observe them… but not only the animals, also the parameters of their environment… the animals react differently.” (ACT2). The animal caretakers from observational studies are clearly more inclined to the bond with animals: “It is necessary for me to have contact with these animals… they come to me… I can pat them… We want to spend time together… like with a human” (ACT6).

### 3.4. Researchers’ Recognition of the Animal Caretakers’ Work

The animal caretakers were recognized as an important part of the research: “The scientists are now conscious that animal caretakers are important… we know the animals… We know how to catch them, how to manipulate them” (ACT6); “During meetings with scientists it was said that without animal caretakers, there would be no animals, and no research… The scientists then acknowledged that we are important, that our job has been evolving… We are better considered, more considered by the hierarchy” (ACT1). All the animal caretakers said that they had good relationships with the researchers and with the laboratory technicians. This, in part, is due to the existence of research ethics committees and animal welfare bodies, and their advice and compulsory recommendations. Animal caretakers actually found the regulations helpful (i.e., ethics committee recommendations, the quality of research regulations, visits from veterinarians for health and welfare purposes). Even although it was the researchers’ responsibility to manage them, it made them aware of the constraints on animal use in research: “I could not imagine not participating in the ethics committee because I think… with people… their different backgrounds and their different positions, you learn extraordinary things, you have discussions which are sometimes heated but they are so interesting…” (ACT6). Researchers considered that the caretakers deserved to be recognized as actors of research, especially given that they may encounter internal conflict due to their bond with animals and the requirement of certain invasive procedures; however, they continue to follow the protocols as much as possible, and they believe in the importance of the research they are participating in: “The animal use has a scientific aim, in order to save other animals or to promote technical or scientific advances” (ACT2); “We are paid for this, it is justified and brings something to research” (ACT5); “A job well done…. it is to leave in the evening feeling that we have accomplished what was asked of us, by following the rules of research and managing the animals welfare…the scientist gets his results and we avoided any pain for the animals” (ACT6). The abattoir worker recognized that, depending on the research objectives, the requirement for the collection of specific organs could vary, and this person attempted to maximize the value of the remaining tissues for other researchers: “If X wants to slaughter a sheep to obtain the digestive tract… if it is a male, I could find someone interested in the testicles… or someone who needed a brain to study”. Caretakers and slaughterhouse workers feel good at work: “We are not only realizing the experimental protocols, but we are also actors” (ACT6); “We have real autonomy in our work, and we have direct contact with the researchers” (ACT1); “We are much better recognized” (ACT3).

## 4. Discussion

Few sociological animal studies have been undertaken [25], and most studies on use of animals in experiments are authored by philosophers. The sociological results presented within this paper are based on interviews from a limited number of stakeholders involved in both experimental and observational research. They do not pretend to present a representative view of all stakeholders; however, the insights provided do maintain legitimacy in the social sciences [19]. The intention was to show how people react in this context rather than why. We aim to make sense of or understand the phenomenon rather than predict or explain it. The study was performed at a single research center, which belongs to a larger national agricultural research institute with a centralized organization structure. We would thereby expect the results to be similar across the different research centers within the same institute. Experimental research institutes within Europe follow the same directives for animal welfare; as such, our conclusions could potentially be generalized across other institutes. This may, however, be different in the field of genetic selection, particularly for mice, where it has been suggested that super-reification may occur [25].

An increasing number of computer software programs aim to support content analysis or qualitative data analysis, with several tools dedicated specifically to text analysis and to the computer-aided interpretation of interviews [23]. The Tropes software [21] offers semantic analysis tools, with graphs based on multivariate analyses, and it is freely available in several languages, including English. Its efficacy, particularly in establishing homogeneous groups of interviewed farmers, has been demonstrated previously [24]. It was again efficient here in categorizing the stakeholders according to their respective positions, including the animal caretakers involved in the research of infectious diseases, the animal caretakers involved in behavioral studies, and the researchers. Based on speech analysis, the Tropes software also highlighted in which respects these groups differed. The word “animal” was used as the center-point for the multivariate analyses, as it was the main interest of this paper and the most commonly used word in the interviews. The graphs clearly showed a difference in the relationship of the words between the stakeholders interviewed (Figure 1). Comparing the frequency with which the words were spoken helped to differentiate the stakeholder’s relationship to the word “animal” and to words such as “team experience”, “animal suffering”, etc. (Table 1). Interpreting interviews is traditionally based on the manual extraction of the sentences of interest. In the absence of any prior categorization of the people interviewed, it can be a delicate task to carry out. Here, by first categorizing the stakeholders into groups using the Tropes software, it was possible to identify, in detail, the views of the stakeholders. We will discuss the most relevant themes based on the information gained by this two-step method: (i) the semi-automatized analyses of the interviews to categorize them and (ii) the extraction of the most characteristic sentences from within these groups.

Animal caretakers were previously reported to have experienced a lack of recognition by researchers and laboratory technicians [12,13] across different research institutes, which may have consequently resulted in a degree of suffering amongst the animal caretakers [9,26,27,28]. However, this has since changed, and this may be attributable to the increased opportunities for continual education in the position and the formal recognition of animal caretakers as professionals [6,26]. In Germany, animal caretakers are required to complete a three-year apprenticeship following their secondary school education. They are involved in several aspects of research, including ethics committee discussions (i.e., the availability of animals, their nutrition, their potential to incur pain, participating in the application of the 3 Rs, etc.). The researchers do not maintain a close daily relationship with the experimental animals, even although they consider animal welfare to be a priority. Furthermore, the researchers are primarily trained as biologists, and at INRAe, they are no longer recruited from a diverse panel of veterinarians or agronomists. They are therefore reliant upon the animal caretakers to maintain the animals in good condition. Animal caretakers have now become an integral part of the research efforts, and their reification by researchers based on their status has largely passed.

Herzog previously stated that “People who work in biomedical and behavioural research settings sometimes form strong relationships with individual laboratory animals. Ethnographic studies indicate that it is common for these individuals to be transformed from “experimental subject” into to a “pet” status.” [28]. We observed this tendency, particularly amongst animal caretakers working in behavioral studies. There are aspects that may oppose this bond, as the caretakers will generally remain in contact with the animals over a very long period of time, and they are not intended for necropsy. This bond may also occur for animals that are considered intelligent and receptive to humans, even when they are not required for long periods, such as for the research of infectious diseases (i.e., the mice and rats in this study). This bond may lead to compassion, but also to compassion fatigue: “Moral stress and emotional dissonance may also arise in personnel who may have entered the occupation because of their love, respect and empathy for animals, as well as their desire to care for them.” [29]. The maintenance of livestock animals for research often does not vary much from the standard husbandry practices of farms. Reification was thus more pronounced in animal caretakers involved in animal husbandry, where the animal is considered a prototype for research. Although the researchers are not in daily contact with the animals, there was no clear indication that they reify the experimental animals. They were, however, highly concentrated on obtaining results (R1, R2, R3, and R4) to publish and considered the experimental animals to be intended for this purpose: “The demands of efficiency are such that reification seems inescapable” [27]. Thus, “by gradually transforming the naturalistic animal; the living, conscious and sentient creature into an analytic, and identifying the former with the latter, modern experimental care exemplifies forgetfulness” [26]. The frequency with which the word “animal” was used was found to be much lower for the researchers than for the animal caretakers. As both occupations present their research to different audiences, they partly share public opinions on experimental animals, i.e., they should be treated well, and the research should have some relevance to advancing knowledge or to have practical applications in animals or humans. Public opinion was interpreted to denote one of the meanings for the word “people” based on the multivariate analyses of the interviews. Public opinion has also been a concern for experts in animal research [30], where an unfriendly public opinion may lead to “some animal researchers feeling a social stigma, and an exclusion on what they do” [26]. In this study, there was only one clear case where the fear of social disapproval was evident from the interviewed stakeholders (the abattoir worker) who preferred to present themself as an ordinary butcher rather than a person who operated within an animal research institute.

Two main forces seemingly act upon animal reification: one negative (the bond with the animal) and one positive (the usefulness of animal research). The animal bond exists to varying degrees, as do views on the utility of the research. The majority of the stakeholders believed that animal research was useful, a finding that was also observed by [29]. Yet, two researchers (R1, R3) questioned the utility of some of the research dedicated to intensive agriculture. Philosophers have noted that the benefits of research are not always obvious, owing to the moral value of the animals [31,32]. The interactions between the bond with the animal and the usefulness of research may lead to complex gradients of animal reification.

For the sake of simplicity, we took the term reification to mean “forgetfulness of recognition”, as stated by Honneth [16]. Reification can be more explicitly described by four experiential types: (a) beliefs and opinions which are not to be discussed; (b) the experience of abstractions as being more real than particular objects; (c) the experience that the means (technology and production) justify the ends, or the ends (life) justify the means; and (d) the experience of suspending genuine emotional engagement [33]. The (a) type corresponds to the belief that the benefits of research are more important than the potential harm it may cause; (b) can relate to the view of some researchers that their specific experimental animals are those under scrutiny from public opinion; (c) may apply to aspects of agricultural research, such as animal husbandry practices in intensive livestock production systems; and (d) may relate to the absence of a bond with the experimental animals. Using the typology of Gunderson [33], we can identify circumstances that may lead to the reification of animals by stakeholders and, in turn, use this information to potentially negate such an outcome. According to the typology of Gunderson [33], a simple suggestion to reduce the reification of animals would be to consider them as patients and not as cases [26]. Harm to the animals would thereby be minimized, and their intrinsic and subjective value acknowledged [18]. Such an outlook has been documented in behavioral research where animal reification was also found to be minimal, which may support the adherence to such a view could translate in practice.

## 5. Conclusions

Concerning the methodology, we found that the multivariate analyses of speech was of great benefit in identifying homogeneous groups. Although the stakeholders were all concerned with animal welfare, differences in the intensity of animal reification were observed between them. The first group, which consisted of animal caretakers involved in behavioral studies, was found to develop a bond with the animals, and thus, animal reification was limited. In the second group, which consisted of animal caretakers involved in the research of livestock infectious diseases and more invasive laboratory procedures of animals over shorter durations, animal reification was found to be more present. The third group, composed of researchers who had less contact with the animals, shared similar views on the animals to those of the general public, and their main focus was on obtaining results from the experiments. Ultimately, animal reification was found to be limited but could be even further reduced if the experimental animals were considered as patients, not just as cases.

## Figures and Tables

**Table 1:** Characteristics of the stakeholders in animal research that were interviewed.

Profession	Background	Gender	Experimental Animal Species	Field of Research
Animal caretaker (ACT) ACT1	Rural	Male	Sheep, Goats, Cattle	Parasitology
ACT2	Urban	Female	Sheep, Goats	Parasitology
ACT3	Rural	Male	Sheep	Production of experimental animals
ACT4	Urban	Female	Mice, Rats	Infectious diseases
ACT5	Rural	Male	Horse	Behavior
ACT6	Urban	Male	Horse	Behavior, Parasitology
Abattoir worker ACT7	Rural	Male	Sheep, Goat, Horse, Cattle	Experimental livestock
Laboratory technician (LT) LT1	Urban	Male	Sheep, Horse	Parasitology
Researcher (R) R1	Urban	Male	Sheep, Goat, Cattle, Horse, Rat	Parasitology
R2	Urban	Male	Mice	Parasitology
R3	Rural	Female	Poultry	Behavior, Nutrition
R4	Rural	Female	Horse	Behavior

**Table 2:** The percent occurrence of words used by different categories of stakeholders in animal research.

Words Employed (in Percent)	Animal Caretakers (Behaviorial Research)	Animal Caretakers (Infectious Diseases)	Laboratory Technicians and Researchers
Animal	56	39	32
People	16	12	19
Researcher/Research	8	8	16
Protocol	7	6	2
Continuing education	5	5	0
Animal husbandry	5	5	0
Work	3	16	11
Team/co-workers	0	4	1
Satisfaction		4	5
Suffering and emotions	0	0	8
Problem	0	1	6
Total number of words	433	899	691

## Data Availability

Not applicable.
